# High dose anakinra for treatment of severe neonatal Kawasaki disease: a case report

**DOI:** 10.1186/1546-0096-12-26

**Published:** 2014-07-11

**Authors:** Ashley Shafferman, James D Birmingham, Randy Q Cron

**Affiliations:** 1 University of Alabama School of Medicine, 1720 2nd Avenue South FOT 1203, Birmingham, AL 35294-3412, UK; 2 Division of Pediatric Rheumatology, Helen Devos Children’s Hospital, 35 Michigan St NE Suite 4150, Grand Rapids, MI 49503, USA; 3 Division of Pediatric Rheumatology, University of Alabama at Birmingham, 1600 7th Avenue South CPP 210, Birmingham, AL 35233-1711, USA

**Keywords:** Kawasaki disease, Macrophage activation syndrome, Secondary hemophagocytic lymphohistiocytosis, Anakinra, Interleukin-1 receptor antagonist, Neonate

## Abstract

We report an 11-week-old female who presented with Kawasaki disease (KD) complicated by macrophage activation syndrome (MAS). The infant presented to the hospital with persistent fever, cough, diarrhea, and emesis, among other symptoms. Her condition quickly began to decompensate, and she developed classic features (conjunctivitis, rash, cracked lips, distal extremity edema) prompting a diagnosis of acute KD. The patient was treated with standard therapy for KD including three doses of intravenous immunoglobulin (IVIG), aspirin, and high dose glucocorticoids with no change in her condition. Due to a high suspicion for MAS, high dose anakinra therapy was initiated resulting in dramatic clinical improvements. She also received one dose of infliximab for concern for coronary artery changes, and over the course of several months, anakinra and high dose glucocorticoids were tapered. Nearly complete reversal of echocardiogram changes were observed after 8 months, and the infant is now off all immunosuppressive therapy. In this case report, we briefly review the importance of early recognition of MAS in pediatric patient populations with rheumatic diseases, and we suggest early initiation of anakinra therapy as a rapid and effective treatment option.

## Background

Kawasaki disease (KD), a condition of unknown etiology, is one of the most common acute inflammatory vasculitides in children and infants. Diagnosis of KD is based on clinical findings, resulting from diffuse inflammation of small and medium sized vessels. The most common acute inflammatory manifestations in KD include fever, bilateral nonexudative conjunctival injection, erythema of oral mucosa, peripheral edema, polymorphous rash, and cervical lymphadenopathy [1]. Though no specific laboratory test exists for the diagnosis of KD, laboratory studies reveal typical findings, including: leukocytosis with neutrophilia, elevated erythrocyte sedimentation rate (ESR), elevated C-reactive protein (CRP), normocytic anemia, hyponatremia, hypoalbuminemia, and thrombocytosis [1,2]. Less commonly seen but associated with KD are elevated transaminases (30%), mild hyperbilirunemia (10%), and sterile pyruia (33%) [3,4]. Early identification and initiation of treatment is critical in improving the outcome of KD. Approximately 15-25% of children left untreated, or with a severe delay in treatment, develop coronary artery aneurysms, which can lead to myocardial ischemia, infarction, and death [5]. Initial treatment of KD involves administration of high dose intravenous immunoglobulin (IVIG) and aspirin. In cases of refractory KD, retreatment with IVIG, corticosteroids, or infliximab, a tumor necrosis factor inhibitor (TNF-i), have been shown to be effective treatment [1,6,7].

A life threatening complication of KD that may easily remain undiagnosed is macrophage activation syndrome (MAS), also known as secondary hemophagocytic lymphohistiocytosis (HLH). Though the pathophysiology is not completely understood, it is thought that MAS results from an uncontrolled immune response to certain immunologic stressors such as infections, malignancies, and autoimmune or autoinflammatory states. Undiagnosed and untreated, MAS results in a systemic inflammatory reaction characterized by excessive cytokine production, activation of macrophages, and hemophagocytosis. Patients diagnosed with MAS have been shown to exhibit decreased natural killer (NK) cell number and function [8-11].

Unfortunately, since many of the clinical and laboratory findings of acute KD, including fever, rash, and elevated transaminases, are shared with MAS, it can be difficult to distinguish between the two processes and requires a high degree of suspicion for diagnosis [1,8]. An important, available, timely, and affordable diagnostic marker of MAS is an elevated serum ferritin level. Other laboratory values highly characteristic of MAS include elevated triglycerides, elevated liver enzymes, and decreased fibrinogen [12]. In a retrospective review of 638 children with acute KD, the 12 (1.9%) children found to also have a diagnosis of MAS all exhibited hyperferritinemia. Hypofibrinoginemia, cytopenia in 2 or more cell lines, hypertriglyceridemia, and hepatosplenomegaly were seen in 11 of the 12 children with concomitant MAS. Treatment beyond the standard protocol for acute KD, IVIG and aspirin, was required in all children except 1 [13]. Recently, several case reports have successfully demonstrated the use of the interleukin-1 (IL-1) receptor antagonist, anakinra, in the treatment of KD (1-2 mg/kg/day) complicated by MAS, as well as other pediatric and adult diseases complicated by MAS [14-18]. In this case presentation, we report the use of high dose (9 mg/kg/day) anakinra in the treatment of severe neonatal KD complicated by MAS.

## Case presentation

An 11-week-old Caucasian female fraternal twin, from non-consanguineous parents who were not from a genetically isolated population, presented to the hospital with two days duration of cough, congestion, rhinorrhea, diarrhea, emesis, decreased oral intake, reduced urine output, and fever of 103 °F. She was admitted for dehydration and begun on IV fluids. Initial laboratory studies revealed neutrophilia, anemia, normal platelet count, hyponatremia, and mild hyperbilirubinemia (Table 1). Urine studies revealed sterile pyuria, and blood cultures were reassuringly negative after 48 hours. Work up for CMV, HHV8, parvovirus B19, adenovirus, and HSV infections were all negative.

**Table 1:** Laboratory values

**Hospital Day**	**CRP, mg/dl (normal <10)**	**ESR, mm/hr (normal <15)**	**Hb, g/dl (normal 12.5-19)**	**WBC × 10**^**3/**^**mm**^**3**^** (normal 6–18)**	**Neutrophil (%) (normal 19–45)**	**PLTs × 10**^**3/**^**mm**^**3**^** (normal 140**–**400)**	**AST, U/l (normal 15–60)**	**ALT, U/l (normal 10–40)**	**Triglyceride, mg/dl (normal <149)**	**Fibrinogen, mg/dl (range 220–450)**	**Ferritin, ng/ml (normal 50–200)**	**GGT, U/l (normal 8–80)**	**Other labs and important events related to clinical course**:
1			9.6	14.3	57	229							Serum sodium 130 mEq/L Total bilirubin 3.5 mg/dL
3	133.4	40	6.9	12.12	59	32	25	18				138	Serum sodium 131 mEq/L Hypoalbuminemia of 1.8 g/dL (reference range: 3.4-5.4 g/dL) IVIG dose #1
4			10.1	16.8		121	24	19	82		801		IVIG dose #2/Transfusion
6	191.6	24	9.9	27.5		66	22	17		239	255		IVIG dose#3 Methylprednisolone 30 mg/kg × 3 days Flow cytometry
7	234.1	113	8.2	16.6		52				273	207		**Anakinra started**
													(3 mg/kg BID) (increased to 3 mg/kg TID after 3 days)
8	122.9	64	7.8	21.5		133	16	14			213		Slight clinical improvement
9	65	37	12.7	22.6		156			165				Methylprednisolone (1 mg/kg BID) Marked clinical improvement Extubated day 9 Tranfusion
12	98.3	67	13.2	19.6		607	32	21	288		574		Echocardiogram: diffuse enlargement of the entire coronary artery system Infliximab 5 mg/kg ×1 Methylprednisolone increased (4 mg/kg TID)
16	1.6	25	11.3	12.9		963	27	28	201	239	700	108	Methylprednisolone decreased (2 mg/kg TID)
20	0.3	5	13	20.9		972			269	212	754	134	Methylprednisolone decreased (2 mg/kg Daily)
27	<0.2	8	11.3	16.7		427			293		406	58	Anakinra decreased (4 mg/kg BID) Methylprednisolone stopped Prednisolone started 1.5 mg/kg Daily (tapered over 10 days)

*CRP*: C-reactive protein; *ESR*: erythrocyte sedimentation rate; *Hb*: hemoglobin; *WBC*: white blood count; *PLTs*: platelets; *AST*: aspartate aminotransferase; *ALT*: alanine aminotransferase; *GGT*: gamma-glutamyl transpeptidase; *IVIG*: intravenous immunoglobulin treatment.

On hospital day 3, the infant developed a diffuse, erythematous, macular-papular blanching rash on the face, trunk, and extremities. She was also noted to have peripheral edema, non-purulent injected bilateral conjunctiva, dry and cracked desquamating rash on her lips and eyelids, mild hepatomegaly, and a few small (<1 cm) bilateral cervical lymph nodes. Progressive respiratory distress developed requiring intubation. Repeat laboratory studies showed persistent neutrophilia, worsening anemia, new onset thrombocytopenia, normal transaminases, mild hyponatremia, hypoalbuminemia, and elevated acute phase reactants (Table 1). A cerebral spinal fluid (CSF) analysis revealed pleocytosis with mononuclear cell predominance, normal glucose, and mildly elevated protein. An echocardiogram showed a right coronary artery diameter at the upper limits of normal with no evidence of aneurysm. Due to a high suspicion for KD, an abdominal ultrasound was performed showing hydrops of the gallbladder with circumferential edematous thickening of the gallbladder wall, further supporting the clinical diagnosis. The patient was subsequently started on aspirin and 2 g/kg IVIG on days 3, 4, and 6 of hospitalization (Table 1). Echocardiogram of the coronary arteries on day 4, revealed the following measurements and associated Z-scores: 0.16 cm (Z - 0.91), 0.17 cm (Z – 1.4), and 0.19 cm (Z – 0.25) for the left anterior descending (LAD), right (RCA), and left main (LM) coronary arteries, respectively.

On day 6 of hospitalization, flow cytometry was obtained due to concern for MAS (elevated ferritin and thrombocytopenia). Results revealed few NK cells (<1%) and decreased CD8 T cells compared to the reference range for age making it difficult to analyze cytolytic function; however, there were normal perforin and granzyme B levels in both cell lines. Genetic analyses detected no mutations in familial HLH genes (STXBP2, STX11, MUNC13-4, RAB27A). Subsequently, the patient was given a three-day course of high-dose methylprednisolone, 30 mg/kg/dose. Despite treatment with IVIG and methylprednisolone, the patient’s conditioned remained unchanged. A repeat echocardiogram was obtained revealing that the right coronary artery and the left anterior descending artery were mildly dilated, and the left main coronary artery was at the upper limits of normal, all significantly larger than on the initial echocardiogram four days prior. The infant was started on therapy with high dose anakinra (3 mg/kg/dose, twice daily for 3 days, then increased to three times a day for rash and rise in CRP). There was an immediate reduction noted in pulmonary resistance by mechanical ventilation, and she stabilized in terms of her vital signs. By day 3 of treatment the patient was displaying a dramatic improvement in her rash and an impressive reduction in her overall inflamed appearance, as well as decreased levels of CRP and an increasing platelet count (Table 1). A second rise in ferritin occurred despite a remarkable rise in platelet count and fall in CRP coincident with anakinra treatment. Perhaps this was a reflection of liver and bone marrow recovery as she dramatically improved clinically.

Despite noted clinical improvements with the use of anakinra and methylprednisolone, a third echocardiogram revealed interval diffuse enlargement of the entire coronary artery system [LAD – 0.36 cm (Z – 11), RCA – 0.32 cm (Z – 6.5), LM – 0.37 cm (Z – 5.6)] without discrete aneurysms. Due to concerns about progressive coronary changes, therapy was supplemented with one dose of infliximab (5 mg/kg) and increased methylprednisolone (4 mg/kg/dose, three times a day). The patient remained clinically stable and slowly tapered off prednisolone (1.5 mg/kg/day) over the 10 days following discharge, and then off anakinra (8 mg/kg/day, divided twice daily) slowly over the next 5 months. A repeat flow cytometry study, approximately 6 months after initial laboratory studies, revealed reduced perforin and granzyme B in CD8 T cells, normal perforin and granzyme B in NK cells, and normal absolute levels of both CD8 T cells and NK cells. An echocardiogram 8 months after initial coronary artery changes revealed normal coronary artery origins, with only mild dilation of the proximal right coronary artery, and a left main coronary artery at the upper limits of normal in size [LAD – 0.18 cm (Z – 0.84), RCA – 0. 24 cm (Z – 2.8), LM – 0.26 cm (Z – 1.6)]. The patient is now off all immune suppressive therapy and is maintained on low dose aspirin and propranolol, with regular follow-up appointments with her cardiologist and rheumatologist.

## Discussion

Our case highlights the importance of anakinra, an IL-1 receptor antagonist, in children with KD complicated by MAS. Our patient was rather young for typical KD and may not be universally representative, but she did meet KD criteria and had evidence of coronary artery abnormalities. Although our patient did not have sCD25 measured and did not meet restrictive familial HLH criteria, she had a >88% chance of having reactive HLH/MAS as per the newly developed HScore [19]. Though only 1.9% of children with acute KD are reported to develop overt MAS, it is unclear whether there is a greater percentage with subclinical MAS as in children with systemic juvenile idiopathic arthritis (sJIA) [13,20]. It is also unknown whether KD complicated by MAS is being labeled as Kawasaki shock syndrome (7% of KD patients studied), refractory KD, or recurrent KD, as the processes may appear to have similar clinical features [21,22]. At present it is unclear whether Kawasaki shock syndrome is on the MAS spectrum or whether a subset of children with Kawasaki shock syndrome have frank MAS. Recently, a retrospective survey was conducted in Korea to determine differentiating factors between recurrent KD and KD complicated by MAS. The study concluded that the most important distinguishing factor was the time course of symptoms and signs. The onset of MAS following KD was approximately 13.3 days (range, 3–22 days); however, recurrent KD typically occurred much later at a mean of 17.9 months (range, 1–60 months) [21].

It is critically important to identify MAS early, as the potentially devastating complication carries a high mortality rate and may require a more aggressive treatment approach than the traditional treatment protocol for KD [23]. The initial treatment strategy for MAS involves supportive therapy and aggressive immunosuppressive treatment to reduce the severe systemic inflammation, which causes the life threatening symptoms. While it is highly important to control hyperinflammation, suppressing the patient’s remaining immune system can also pose significant risks. Traditionally, immunosuppressive therapies used in the treatment of MAS include IVIG, high dose glucocorticoids, and cyclosporine [24]. Etoposide has also been suggested for treatment of MAS but carries high morbidity and mortality rates [25].

As an alternative to traditional therapy for MAS, we suggest a more targeted therapy with anakinra as an effective and rapid treatment for MAS in children and very young infants. Additionally, our case presentation demonstrates that anakinra used with infliximab can be an effective combination therapy in treating MAS and KD associated complications. Several case reports have shown successful use of the IL-1 receptor antagonist, anakinra, in treating children with various rheumatologic conditions complicated by MAS [14-16]. In addition to treating a case of KD associated MAS [15], low dose (1-2 mg/kg/day) anakinra has also been shown to successfully treat refractory KD [26]. Because infants with autoinflammatory disorders [27] and cases of severe MAS may require higher dosing of anakinra [14], it may be prudent to treat with higher doses of anakinra until the inflammation can be controlled. Recently, a retrospective case series demonstrated the therapeutic role of anakinra in 8 children in the intensive care unit that were diagnosed with secondary HLH or MAS. The study showed promising results with dramatic decreases in ferritin, fibrinogen, and CRP levels following initiation of anakinra. Importantly, no infections were attributed to the use of anakinra [18]. Infliximab may also play a role in lowering inflammation in KD and potentially reducing coronary artery abnormalities [28,29].

## Conclusions

It appears that early intervention with high dose anakinra may provide a safe and effective alternative to treatment of life-threatening MAS in children. As further knowledge of MAS, or secondary HLH, comes to the forefront, it is imperative to consider this diagnosis in critically ill children, especially those with underlying rheumatic conditions. Further study of anakinra in the treatment of MAS, as well as combination therapy of anakinra and infliximab, as a therapeutic alternative in KD associated MAS with coronary artery dilation is warranted.

## Consent

Written informed consent was obtained from the patient’s parents for publication of this Case Report. A copy of the written consent is available for review by the Editor-in-Chief of this journal.

## Abbreviations

CRP: C-reactive protein; ESR: Erythrocyte sedimentation rate; HLH: Hemophagocytic lymphohistiocytosis; IL-1: Interleukin 1; IVIG: Intravenous gamma-globulin; KD: Kawasaki disease; LAD: Left anterior descending; LM: Left main; MAS: Macrophage activation syndrome; NK: Natural killer cell; RCA: Right coronary artery; sJIA: Systemic juvenile idiopathic arthritis; TNF-i: Tumor necrosis factor inhibitor.

## Competing interests

The authors declare that they have no competing interests.

## Authors’ contributions

JB collected and reviewed the data. AS and JB drafted the manuscript. RQC and JB contributed to the interpretation of the patient data. RQC helped draft the manuscript. JB and RQC were involved in the patient care. JB and RQC formatted the table and contributed to the conception and design of the study. All authors critically revised the manuscript and read and approved the final manuscript.
